# Analysis of early diagnostic pathway for prostate cancer in Slovenia

**DOI:** 10.2478/raon-2024-0046

**Published:** 2024-10-04

**Authors:** Mateja Kokalj Kokot, Spela Mirosevic, Nika Bric, Davorina Petek

**Affiliations:** Department of Family Medicine, Faculty of Medicine, University of Ljubljana, Ljubljana, Slovenia; Primary Healthcare Centre Grosuplje, Grosuplje, Slovenia; Sector for Oncology Epidemiology and Cancer Registry, Institute of Oncology Ljubljana, Ljubljana, Slovenia; Medical Centre Zdravje, Ljubljana, Slovenia

**Keywords:** prostate cancer, doctor interval, primary care interval

## Abstract

**Background:**

Prostate cancer (PCa) is a prevalent male malignancy globally. Prolonged diagnostic intervals are associated with poorer outcomes, emphasizing the need to optimize this process. This study aimed to evaluate the doctor and primary care interval, research their impact on patient survival and explore opportunities to improve PCa diagnostic pathway in primary care.

**Patients and methods:**

A retrospective cohort study using cancer patients' anonymised primary care data and data of the Slovenian Cancer Registry.

**Results:**

The study found that the doctor interval had a median duration of 0 days (interquartile range ([IQR] 0–6) and primary care interval a median duration of 5 days (IQR 0–58). Longer intervals were observed in patients with more than two comorbidities, where general practitioners didn't have access to laboratory diagnostic tests within their primary health care centre and when patients first presented with symptoms (reported symptoms at first presentation: dysuria, lower urinary tract symptoms [LUTS], abdominal pain). The analysis also revealed a statistically significant association between lower 5-year survival rate and the accessibility of laboratory and ultrasound diagnostics in primary healthcare centres and a shorter 5-year survival of symptomatic patients in comparison to patients who were identified by elevated levels of prostate specific antigen (PSA).

**Conclusions:**

This study shows that treating suspected PCa in primary care has a significant impact on 5-year survival. Several factors contribute to better survival, including easy access to laboratory and abdominal ultrasound in primary care centres. The study highlights the complex array of factors shaping PCa diagnosis, beyond individual clinicians' skills, encompassing test and service availability.

## Introduction

Prostate cancer is a common malignant tumour and has the highest incidence of all non-cutaneous cancers worldwide in males. It is also the fifth leading cause of cancer death among men in 2020.^1^ In Slovenia the 2020 age-standardized (World standard population) incidence rate was 62.7.^2^ In Central and Eastern Europe it was 46.4 and 59.1 in Southern Europe.^1^ The ageing population undoubtedly influences the increasing incidence of prostate cancer. However, the proportional growth of elderly individuals (aged 65 years and older) in Slovenia has not escalated sufficiently to solely account for this rise in prostate cancer incidence. For example, in 1991, the proportion of elderly people in Slovenia was 11.2% and the crude incidence rate for prostate cancer was 26.3 per 100,000 individuals. In 2001, the proportion of elderly increased to 14.3% and the crude incidence rate for prostate cancer was 74.5.^3,4^ The dramatic increase in the incidence of prostate cancer over the past two decades is not due to any newly identified risk factor, but rather to the increasing use of the prostate-specific antigen (PSA) test in healthy men and therefore detection of a large number of cancers that would have otherwise remained undetected for life. Data for recent years indicate that we have already reached the peak incidence of prostate cancer.^2^ The histological incidence far surpasses the proportion of individuals in whom the disease is manifested, and while there has always been a tendency not to misdiagnose those with prostate cancer, there is increasing focus on identifying patients who are treated by watchful waiting. Prostate cancer is characterised by a slow natural course of the disease, with the majority of patients dying from other, non-cancer related causes.^5,6^ Both the incidence and mortality rates of prostate cancer in Slovenia are above the European average. In 2020, Slovenia reported an age-standardized (World standard population) mortality rate of 14.9, while Central and Eastern Europe recorded a rate of 13.7, the highest among all European regions.^1,2^

Much effort has already been applied to optimize early detection in prostate cancer and some medical professional societies and organizations recommend prostate cancer screening or discussing screenings with men of suitable age and life expectancy.^7^ Many countries in Europe have joined PRAISE-U project to establish screening programme for prostate cancer.^8^ In primary care-based health care systems, in which the general practitioner (GP) is the patient's first contact and triages the patient's further access to the system, most prostate cancer patients either present to a GP with symptoms (dysuria, lower urinary tract symptoms (LUTS), abdominal pain) or an elevated level of PSA is found in asymptomatic patients. Therefore, timely recognition of cancer-related complaints and adequate referral by the GP are and will remain essential to reduce time to diagnosis until successful screening programmes are introduced. Even though the association between time intervals in the diagnostic pathway and clinical outcomes is complex and remains debated, evidence suggests an association between shorter times to diagnosis and more favourable outcomes in breast cancer, colorectal, head and neck, testicular cancer and melanoma.^9,10^ Optimising the diagnostic pathway from first presentation to diagnosis and start of treatment, usually interpreted as shortening the diagnostic phase, has therefore been a main objective of health care organisations involved in cancer care worldwide. For some countries in Europe, the duration of several of these intervals has been charted.^11,12,13,14,15,16,17^ For other countries, such as Slovenia, the duration of these intervals is unknown. International comparison of the duration of diagnostic intervals in different health care systems and cultural environments is important to identify system-, disease- and patient-related factors that contribute to an unnecessarily prolonged patient journey. Exploring the duration of the diagnostic pathway in Slovenia and how primary care contributes to it generates relevant information on international differences in the duration of the diagnostic pathway. This provides the opportunity to distinguish underlying mechanisms of delay, including system-, disease- and patient-related delay.

The aims of the study were (i) to evaluate the duration and specifics of management during the doctor and primary care intervals in Slovenia, (ii) to investigate the potential association between the durations of the intervals, sociodemographic and organizational variables and (iii) to assess the association between these variables, intervals and the 5-year survival rates of prostate cancer patients in Slovenia.

## Patients and methods

### Study design, data source and patient selection

A retrospective cohort study was performed using data from the Slovenian Cancer Registry and primary care data collected from selected GPs and family doctors with whom these patients were registered at the time of the study. Cancer Registry of Republic of Slovenia provides reliable and detailed information on Slovene cancer patients since 1950. This study was part of the research project of the Institute of Oncology Ljubljana, in collaboration with the Department of Family Medicine, Faculty of Medicine, University of Ljubljana, and the Clinical Department of Urology, University Medical Centre Ljubljana, entitled Integrated analysis of the early management of patients with urological cancers, assessing delays in referral, diagnosis and first treatment. The study was reviewed and approved by the Commission of the Republic of Slovenia for Medical Ethics (0120-233/2019/4).

**FIGURE 1.**
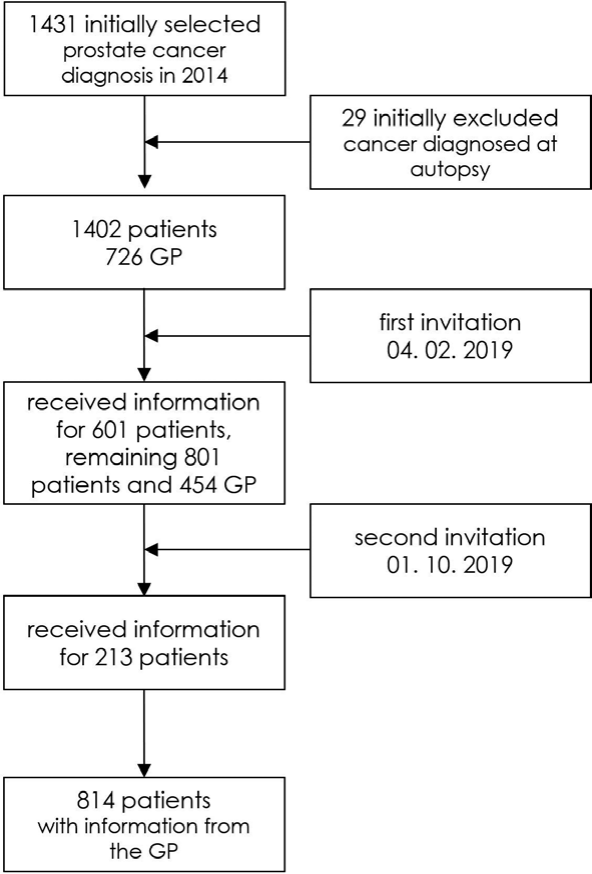
Flowchart of patient selection. GP = general practitioner

We included all newly diagnosed prostate cancer patients during the year 2014 in the Slovenian Cancer Registry database. Consecutively, 1431 patients with all stages of prostate cancer were included. We then linked the Cancer Registry data to the National Insurance Company Registry in order to identify each patient's GP and send them a questionnaire. The exclusion criterion was if the diagnosis of prostate cancer was made at the autopsy (29 patients). A flowchart of patients' data inclusion in our study is presented in Figure 1. We asked the GPs to collect information from the patient's records and complete the attached questionnaire on management of the initial symptoms of the disease, the diagnostics performed and referral decisions, using the dates and information given in the patients' medical record and discharge letters from hospitals. We also asked them about the accessibility to diagnostics (laboratory, abdominal ultrasound) in their primary healthcare centre. Non-responders received a reminder after eight months.

### Data collection

#### Doctor interval and primary care interval

The intervals were defined according to the Aarhus statement.^18,19^ Doctor interval was defined as the period of time from first consultation in primary care to beginning of the first investigation in primary care. Date of first presentation was defined as the first contact with the GP (in person or telemedicine) with possible prostate cancer related signs and/or symptoms. The questionnaire allowed the GP to choose from six different reasons why the patient chose to see a doctor, with multiple choices also possible. Possible symptoms and signs reported by the patient at first presentation were dysuria, LUTS and lower abdominal pain. Additional reasons for visiting the doctor included a family history of cancer, an elevated PSA level detected during routine check-ups (preventive examination, opportunistic screening, regular annual follow-up for chronic diseases, occupational health and safety check-up), and doctors could also choose other. Those were the cases where prostate cancer was not first suspected in the GP's practice but elsewhere, e.g. during hospitalisation for another illness, as reported by the GPs in the questionnaire notes. GPs themselves set the most accurate date estimate for the first presentation after reviewing the patient's records.

They also noted if and when they performed a digital rectal exam, referred the patient to the abdomen ultrasound and/or checked the patient blood (complete blood count [CBC] and/or C-reactive protein [CRP] and/or PSA) and/or urine. All of the stated actions counted as first investigation in primary care. We calculated the length of the doctor interval based on the date of the first presentation and the date of the earliest diagnostic procedure.

Primary care interval was defined as the period of time from first presentation in primary care to referral to urologist. Date of referral was defined as the day the referral letter to the urologist was written, which was also stated by the GP.

If the date of first presentation was the same or later than the date of the earliest diagnostic procedure, the duration of doctor interval was coded as 0 days. Similarly, if the date of first presentation was the same as the date of the referral to urologist, the duration of primary care interval was coded as 0 days. If the length of any interval was equal to or more than one year, it was coded as 365 days.

#### Characteristics

The decision to collect data for certain characteristics and to include them in our analyses was based on previously reported diagnostic procedures and predictors in the literature^20,21,22,23,24,25^, on clinical relevance of patients and disease characteristics, and on availability of data in the Cancer registry and primary care data. The questionnaire was approved by the entire research team. We collected data on age, level of education, stage of cancer, comorbidities, symptoms and signs at first presentation, vital status 5 years after diagnosis and location of their GP's primary health care centre.

The Slovenian Cancer Registry Database uses a simplified definition of stages at diagnosis for solid tumours, classifying them into localized, spread (regional) and metastatic stage of disease. The simplified stage definition generally follows the TNM classification. Localized stage includes all cancers where the tumour has been classified as T1 and T2. In these cases, neither regional lymph node involvement nor distant metastases are found (N0, M0). The spread stage includes tumours classified as T3 and T4 and/or with regional lymph node metastases (N1), without presence of metastases in distant lymph nodes or organs (M0). A disease with metastases in distant lymph nodes or organs is classified as a metastatic stage (M1).^2^

### Statistical analysis

The data on the patients' demographics, cancer stage, symptoms at first presentation, comorbidities, diagnostics executed at the primary health care centre, region of GP's practice and accessibility to diagnostic tests reported in questionnaires were collected and managed in Microsoft Access Database 2007–2016 Version. Additional data processing was performed in Excel (Microsoft Office Professional Plus 2019). Descriptive statistics, including means, median, standard deviations and frequencies, were calculated to provide an overview of the data distribution. To examine the relationships between variables, we used Independent Sample T-test and chi-square tests for categorical variables. In case of the presence of variables with non-normal distributions, we also used the Mann-Whitney U-test. To identify potential predictors for the outcome variable, we performed a multiple logistic regression analysis that included the relevant sociodemographic, clinical and organizational variables.

To account for missing data that occurred randomly and without a recognizable pattern, we conducted multiple imputation and analysis using Version 29.0 of the IBM SPSS Statistics. This technique allows the estimation of missing values by creating multiple plausible imputation datasets, thereby maintaining statistical power, and minimizing bias.

All statistical analyses were performed using IBM SPSS Statistics with the significance level set at p < 0.05.

## Results

### Patients' characteristics

Among the cohort of 1431 patients registered with a diagnosis of prostate cancer in the Cancer Registry in 2014, we successfully obtained primary care data for a total of 814 patients, as illustrated in Figure 1.

The included patients' average age was 69 years, with a standard deviation of 8.5 years. Detailed sociodemographic and clinical characteristics of the prostate cancer patients included in this study are presented in Table 1.

**TABLE 1.** Sociodemographic and clinical characteristics of prostate cancer patients (N = 814)

**Characteristics**	**Sample of prostate cancer patients, n (%)**
Age (mean ± SD), range: 44–97	69.0 ± 8.5
**Age, groups**	
< 65	265 (32.5)
65–75	357 (43.9)
> 75	192 (23.6)
**Cancer stage**	
Localized	572 (70.3)
Spread	191 (23.4)
Metastatic	51 (6.3)
**Education**	
Primary/Elementary education	280 (34.4)
Secondary/High school education	386 (47.4)
Higher education	148 (18.2)
**Comorbidities**	
No. of comorbidities (mean ± SD), range: 0–6	1.2 ± 1.2
None	290 (35.6)
1–2	410 (50.4)
> 2	114 (14)
**Number of patients by region of the primary health care centre's location**	
Pomurska	106 (13)
Podravska	114 (14)
Koroška	31 (3.8)
Savinjska	85 (10.4)
Zasavska	3 (0.4)
Spodnjeposavska	25 (3.1)
JV Slovenija	53 (6.5)
Osrednjeslovenska	204 (25.1)
Gorenjska	69 (8.5)
Notranjsko-kraška	27 (3.3)
Goriška	56 (6.9)
Obalno-kraška	41 (5.0)
**Symptoms and signs at first presentation (multiple options possible)**	
Dysuria	111 (13.6)
LUTS	307 (37.7)
Pain in the abdomen	63 (7.7)
Family history of cancer	17 (2.1)
Elevated PSA	246 (30.2)
Other	130 (16)
**Alive 5-years after diagnosis**	610 (74.9)

JV Slovenija = South-East Slovenia; Koroška = Carinthia; LUTS = lower urinary tract symptoms; Obalno-kraška = Coastal-Karst; Osrednjeslovenska = Central Slovenia; PSA = prostate specific antigen

### Doctor and primary care intervals' characteristics

The median duration of the doctor interval, in terms of days, was found to be 0, with an interquartile range spanning from 0 to 6 days. The median duration of the primary care interval was 5 days, with an interquartile range extending from 0 to 58 days. The average number of visits to the doctor during the primary care interval was 1.68 (SD 0.9), range 1–5. Table 2 provides also the mean of the intervals.

**TABLE 2.** Duration of doctor and primary care interval

	**mean (± SD)**	**range**
**Doctor interval, days**	37.5 (92.8)	0–365
**Primary care interval, days**	67.4 (123.8)	0–365

For the analysis of the doctor and primary care intervals, we excluded the Zasavska region due to a limited sample size, with only three patients included in the study, which did not provide sufficient statistical power for meaningful analysis. Consequently, our analysis was based on a dataset consisting of 811 patients. Table 3 shows the diagnostic procedures performed by the GP and their access to laboratory and abdominal ultrasound within the primary health care centre, overall and by region.

**TABLE 3.** Diagnostic procedures performed by the general practitioner (GP) and their access to laboratory and abdominal ultrasound within the primary health care centre, overall and by region (N = 811)

**Diagnostic procedures performed by GPs**	**Sample of prostate cancer patients, n (%)**				
CBC, CRP			497 (61.1)		
PSA			664 (81.6)		
Urine			471 (57.9)		
US			434 (53.3)		
DRE			255 (31.3)		
**Diagnostic procedures performed by GPs by region**	**CBC, CRP**	**PSA**	**urine**	**US**	**DRE**
Pomurska (N = 106)	48 (45.3)	77 (72.6)	52 (49.1)	39 (36.8)	34 (32.1)
Podravska (N = 114)	77 (67.5)	95 (83.3)	60 (52.6)	68 (59.6)	23 (20.2)
Koroška (N = 31)	15 (48.4)	24 (77.4)	13 (41.9)	4 (12.9)	11 (35.5)
Savinjska (N = 85)	55 (64.7)	64 (75.3)	61 (71.8)	50 (58.8)	26 (30.6)
Spodnjeposavska (N = 25)	14 (56)	20 (80)	16 (64)	11 (44)	13 (52)
JV Slovenija (N = 53)	35 (66)	39 (73.6)	25 (47.2)	19 (35.8)	11 (20.8)
Osrednjeslovenska (N = 204)	135 (66.2)	180 (88.2)	131 (64.2)	140 (68.6)	46 (22.5)
Gorenjska (N = 69)	48 (69.6)	63 (91.3)	44 (63.8)	39 (56.5)	22 (31.9)
Notranjsko-kraška (N = 27)	21 (77.8)	23 (85.2)	19 (70.4)	20 (74.1)	15 (55.6)
Goriška (N = 56)	28 (50)	45 (80.4)	29 (51.8)	22 (39.3)	33 (58.9)
Obalno-kraška (N = 41)	19 (46.3)	32 (78)	19 (46.3)	20 (48.8)	19 (46.3)
**Accessibility to diagnostic tests in the primary health care centre**	**Sample of prostate cancer patients, n (%)**				
Laboratory (CBC, CRP, urine)	734 (90.2)				
Laboratory (PSA)	653 (80.2)				
US	306 (37.6)				
**Accessibility to diagnostic tests in the primary health care centre by region**	CBC, CRP, urine		PSA	US	
Pomurska (N = 106)	96 (90.6)		79 (74.5)	34 (32.1)	
Podravska (N = 114)	98 (86)		89 (78.1)	42 (36.8)	
Koroška (N = 31)	28 (90.3)		26 (83.9)	22 (71)	
Savinjska (N = 85)	78 (91.8)		63 (74.1)	36 (42.4)	
Spodnjeposavska (N = 25)	23 (92)		16 (64)	10 (40)	
JV Slovenija (N = 53)	46 (86.8)		42 (79.2)	15 (28.3)	
Osrednjeslovenska (N = 204)	187 (91.7)		173 (84.8)	74 (36.3)	
Gorenjska (N = 69)	63 (91.3)		60 (87)	31 (44.9)	
Notranjsko-kraška (N = 27)	24 (88.9)		20 (74.1)	7 (25.9)	
Goriška (N = 56)	53 (94.6)		52 (92.9)	23 (41.1)	
Obalno-kraška (N = 41)	36 (87.8)		31 (75.6)	12 (29.3)	

CBC = complete blood count; CRP = C-reactive protein; DRE = digital rectal exam; GP = general practitioner; JV Slovenija = South-East Slovenia; Koroška = Carinthia; Obalno-kraška = Coastal-Karst; Osrednjeslovenska = Central Slovenia PSA = prostate specific antigen; US = abdominal ultrasound

Table 4 presents the median and interquartile range data for the duration of the doctor and primary care intervals, computed based on patient and presentation characteristics.

**TABLE 4.** The distribution and association of selected characteristics with doctor and primary care interval. (N = 811)

	**N**	**%**	**Doctor Interval**			**Primary care interval**		

			**Median**	**Interquartile range**	**P**	**Median**	**Interquartile range**	**P**
**Age groups**					0.131			0.382
< 65	264	32.55	0	0–4		5	0–65	
65–75	355	43.77	0	0–5		4	0–51	
> 75	192	23.67	0	0–20		7	0–73	
**Education**					0.437			0.204
Primary/Elementary education	278	34.28	0	0–6		7	0–38	
Secondary/High school education	385	47.47	0	0–5		3	0–53	
Higher education	148	18.25	0	0–18		7	0–127	
**Region of the location of primary health care centre**					0.863			0.021
Pomurska	106	13.07	0	0–18		3	0–62	
Podravska	114	14.06	0	0–20		8	0–50	
Koroška	31	3.82	0	0–20		0	0–42	
Savinjska	85	10.48	0	0–3		0	0–28	
Spodnjeposavska	25	3.08	0	0–10		3	0–91	
JV Slovenija	53	6.54	0	0–7		1	0–62	
Osrednjeslovenska	204	25.15	0	0–5		10	0–88	
Gorenjska	69	8.51	0	0–2		4	0–54	
Notranjo-kraška	27	3.33	0	0–7		6	0–127	
Goriška	56	6.90	0	0–5		4	0–34	
Obalno–kraška	41	5.06	0	0–20		4	0–48	
**Cancer stage**					0.037			0.058
Localized	569	70.16	0	0–8		6	0–65	
Spread	191	23.55	0	0–1		1	0–28	
Metastatic	51	6.29	0	0–38		3	0–336	
**Comorbidities**					0.001			0.026
None	288	35.51	0	0–31		7	0–101	
1–2	409	50.43	0	0–4		5	5–44	
> 2	114	14.06	0	0–1		0	0–33	
**GP access to laboratory (CBC, CRP, urine)**					< 0.001			< 0.001
Yes	732	90.26	0	0–4		3	0–37	
No	79	9.74	40	0–330		365	0–365	
**GP access to laboratory (PSA)**					< 0.001			< 0.001
Yes	651	80.27	0	0–2		3	0–37	
No	160	19.73	0	0–177		21	0–365	
**GP access to US**					0.781			0.124
Yes	306	37.73	0	0–5		4	0–31	
No	505	62.27	0	0–7		5	0–78	
**Symptomatic patient**					< 0.001			0.001
Yes	399	49.20	0	0–12		5	0–42	
No	219	27.00	0	0–0		0	0–18	

CBC = complete blood count; CRP = C-reactive protein; DRE = digital rectal exam; GP = general practitioner; JV Slovenija = South-East Slovenia; Koroška = Carinthia; Obalno-kraška = Coastal-Karst; Osrednjeslovenska = Central Slovenia; PSA = prostate specific antigen; US = abdominal ultrasound

Statistically significant longer doctor intervals were observed in patients who had localized vs. metastatic cancer stage, presence of more than two comorbidities, where GPs' didn't have access to laboratory diagnostic tests (CBC, CRP, urine, PSA) within their primary health care centre and when patients first presented with symptoms (reported symptoms at first presentation: dysuria, LUTS, abdominal pain).

The primary care interval was also statistically significantly longer in patients who had more than two comorbidities, where GPs' didn't have access to laboratory tests within their primary health care centre and when patients first presented with symptoms. In addition, there was a marked difference between the Osrednjeslovenska and Savinjska regions, with the former having the longest and the latter the shortest primary care interval.

To further elucidate the relationship between the predictor variables and the primary care interval, we employed a logistic regression model (Table 5). We set the limit at a primary level interval duration of 14 days. Omnibus tests of the model coefficients yielded a chi-square statistic of 53.642 at 11 degrees of freedom, resulting in a p-value of less than 0.001. This indicates robust overall significance of the prediction model, confirming that the independent variables significantly contribute to the variability in the primary care interval. Nevertheless, the Nagelkerke R^2^ coefficient indicates that only 9.4% of the variance of the dependent variable is explained by the model, reflecting the complexity of health service utilization behaviour. The model's -2 log-likelihood of 1018.153 indicates a satisfactory fit to the empirical data.

**TABLE 5.** Logistic regression model on predicting primary care interval

**Variables in the Equation**	**B**	**Wald**	**Exp(B) (95% CI)**
Age	−0.004	0.144	0.996 (0.979, 1.015)
Education primary vs secondary	−0.359	4.223	0.698 (0.496, 0.984)^*^
Education primary vs higher	0.145	0.452	1.156 (0.757, 1.765)
Cancer stage localized vs spread	−0.336	3.315	0.714 (0.497, 1.026)
Cancer stage localized vs metastatic	−0.316	0.955	0.729 (0.387, 1.374)
No comorbidities	−0.070	1.051	0.932 (0.815, 1.066)
First symptom - dysuria	−0.175	0.592	0.840 (0.538, 1.310)
First symptom – LUTS	−0.084	0.228	0.919 (0.650, 1.300)
First symptom – abdominal pain	0.132	0.222	1.141 (0.659, 1.977)
First symptom – family history of cancer	0.257	0.249	1.293 (0.471, 0.913)
First symptom – elevated PSA	−0.486	5.804	0.615 (0.414, 0.913)^*^
Accessible US diagnostics	−0.160	0.940	0.852 (0.616, 1.178)
Accessible laboratory diagnostics (PSA)	−0.196	0.660	0.822 (0.512, 1.319)
Accessible laboratory diagnostics (CBC, CRP, urine)	−1.139	11.375	0.320 (0.165, 0.620)^***^
Constant	1.483	4.119	4.405^*^

CBC = complete blood count; CRP = C-reactive protein; LUTS = lower urinary tract symptoms; PSA = prostate specific antigen; US = abdominal ultrasound

* p < 0.05,

** p < 0.01,

*** p < 0.001

### 5-year survival analysis

In the group of 814 prostate cancer patients, 610 (74.9%) were alive five years after diagnosis.

Our analysis showed statistically significant differences in 5-year survival depending on the accessibility of laboratory and ultrasound diagnostics in primary health care centres (Table 6). In addition, we observed lower 5-year survival in symptomatic patients.

**TABLE 6.** 5-year survival in relation to sociodemographic and organizational variables

**Variables**	**Dead n (%)**	**Alive n (%)**	**P value**
**Age groups**			< 0.001
< 65	32 (12)	233 (88)	
65–75	72 (20.2)	285 (79.8)	
>75	100 (52)	92 (48)	
**Education**			< 0.001
Primary/Elementary education	91 (32.5)	189 (67.5)	
Secondary/High school education	84 (21.8)	302 (78.2)	
Higher education	29 (19.6)	119 (80.4)	
**Region of the location of primary health care centre**			NS
Pomurska	32 (30.2)	74 (69.8)	
Podravska	34 (29.8)	80 (70.2)	
Koroška	9 (29)	22 (71)	
Savinjska	18 (21.2)	67 (78.8)	
Spodnjeposavska	5 (20)	20 (80)	
JV Slovenija	15 (28.3)	38 (71.7)	
Osrednjeslovenska	44 (21.6)	160 (78.4)	
Gorenjska	17 (24.6)	52 (75.4)	
Notranjo-kraška	6 (22.2)	21 (77.8)	
Goriška	14 (25)	42 (75)	
Obalno-kraška	10 (24.4)	31 (75.6)	
**Cancer stage**			< 0.001
Localized	124 (21.7)	448 (78.3)	
Spread	36 (18.8)	155 (81.2)	
Metastatic	44 (86.3)	7 (13.7)	
**Comorbidities**			< 0.001
None	72 (24.8)	218 (75.2)	
1–2	86 (21)	324 (79)	
>2	46 (40.4)	68 (59.6)	
**GP access to laboratory (CBC, CRP, urine)**			< 0.001
Yes	159 (21.7)	575 (78.3)	
No	45 (56.2)	35 (43.8)	
**GP access to laboratory (PSA)**			< 0.001
Yes	136 (20.8)	517 (79.2)	
No	68 (42.2)	93 (57.8)	
**GP access to US**			< 0.05
Yes	62 (20.3)	244 (79.7)	
No	142 (28)	366 (72)	
**Symptomatic patient**			< 0.001
Yes	103 (25.8)	296 (74.2)	
No	29 (13.2)	190 (86.8)	

CBC = complete blood count; CRP = C-reactive protein; DRE = digital rectal exam; GP = general practitioner; JV Slovenija = South-East Slovenia; Koroška = Carinthia; NS = not statistically significant; Obalno-kraška = Coastal-Karst; Osrednjeslovenska = Central Slovenia; PSA = prostate specific antigen; US = abdominal ultrasound

There were statistically significant differences between the duration of primary care interval and 5-year survival (204 dead patients, mean primary care interval 90.18 and 610 alive patients with mean interval 59.79, p-value = 0.007).

## Discussion

This study provides an overview of early management of patients with prostate cancer in family medicine in Slovenia. Our aim was to evaluate the treatment timelines and specificities within the doctor and primary care interval. The median length of the doctor interval was very short at 0 days, the primary care interval exhibited a median duration of 5 days. Our study demonstrated statistically significant correlation of cancer stage, comorbidities, and the accessibility of laboratory tests within primary health care centres and survival of prostate cancer patients. The presence of laboratory tests within primary health care centres emerged as a significant determinant of the primary care interval's duration and 5-year survival.

The exclusion of 617 patients' of 1431 patients total from our analysis was necessitated by various reasons outlined in Figure 1. It is worth noting that the proportion of cases excluded due to missing interval data was similar in our study to previous studies.^11^

Evidence about the length of the doctor interval in patients with different cancers is sparse. We found a study of Denmark's cancer patients^26^, where median duration of doctor interval for prostate cancer patients was 0 days and interquartile range (IQR) 0–6 days, which is the same as in our study. One of the reasons for a short doctor interval is that in primary-level diagnostic procedures, doctors sometimes use laboratory and ultrasound tests already performed prior to this first visit (e.g. blood and urine taken from the patient a month earlier, or an abdominal ultrasound performed three months earlier - doctor's interval is negative, marked as 0 days in the analysis). In this way they omit unnecessary duplication of tests and expedite further management of suspected prostate cancer.

There were no statistical differences in the duration of doctor interval between different age groups, levels of education, region of primary health care centre and accessibility to abdominal ultrasound in the primary health care centre. The duration was statistically significant longer when patients had no comorbidities, when they first presented with symptoms in comparison to elevated PSA value and where there was not access to laboratory diagnostics (CBC, CRP, urine, PSA) in primary health care centre. We interpret these results by assuming that multimorbid patients see their doctor more often, for different reasons, and thus are more likely to report or be asked about different symptoms and signs, as opposed to patients without comorbidities. In patients with elevated PSA levels in the blood, the guidelines recommend that prostate cancer should be excluded, so the decision to refer to a urologist was easy and quick. Dysuria, LUTS and abdominal pain are typical symptoms of a number of different diseases and are not in themselves of great predictive value for a cancer diagnosis.^27,28,29^ Men experiencing urinary problems were more inclined to seek medical attention compared to asymptomatic men, leading to more frequent PSA testing and consequently earlier detection of prostate cancer. Our study focused on diagnosing prostate cancer cases in 2014 rather than estimating the overall prevalence that year. Therefore, it's probable that symptomatic cases outnumber asymptomatic ones. In Slovenian primary health care centers, PSA testing is more commonly conducted for symptomatic men or as part of annual check-ups for those undergoing therapy for benign prostatic hyperplasia, rather than for routine screening of asymptomatic individuals. Interestingly, we found that patients with stage spread cancer had the shortest doctor interval, while patients with metastatic cancer had the longest. This may be due to non-specific or absent symptoms and signs in localised prostate cancer. Symptoms and signs become more frequent and pronounced in spread stage of cancer and those caused by metastatic cancer could also be wrongly attributed to other patient's comorbidities. Considering health system factors, international comparisons suggests that the problem of early cancer diagnosis is ubiquitous across contemporary health systems, including high-income countries, though the same underlying problem is manifested differently depending on health service organisation, healthcare professional cultures, and the public understanding of cancer.^10^ This is the first study using Slovenian data to show a correlation between not having easy access to laboratory diagnostics (CBC, CRP, urine, PSA) in primary health care centres, longer duration of doctor and primary care interval and worse 5-year survival. Considering the impact of the length of the intervals on 5-year survival, we assume that the longer intervals are indicative of the broader health care situation (the influence of the patient, the doctor and the local health care system characteristics), which as a whole influenced the survival of this patient.

The duration of the primary care interval has been investigated in several studies.^11,13,14,17,30,31^ The median duration of the primary care interval in our study was 5 days, with an interquartile range (IQR) extending from 5 to 58 days which is shorter than in Helsper et al study, where the median was 14 days (IQR 3-153).^15^ Lyratzopoulos et al^11^ reported the mean length of primary care interval in prostate cancer patients 31 days (25^th^ Centile 2 days, 90^th^ Centile 74 days) whereas in our study the mean was 67.4 days with SD 123.8. The duration was, similar to doctor interval, statistically significant longer when patients had no comorbidities, when they first presented with symptoms in comparison to elevated PSA value and where there was no direct access to laboratory diagnostics (CBC, CRP, urine, PSA) in primary health care centres. Surprisingly, we observed a significant difference between the Osrednjeslovenska and Savinjska regions, with the latter showing a shorter primary care interval. This was the only parameter that showed difference in the Slovenian regions. Osrednjeslovenska region is the most densely populated, the largest in terms of number of inhabitants and the second largest in terms of area. In 2014 the Savinjska region had less than half as many inhabitants as the Osrednjeslovenska region and had the lowest proportion of the population aged over 80 years in the country (4.3%). Osrednjeslovenska region had the highest gross domestic product (GDP) per capita in the country (25.329 €/resident), 11.7% registered unemployment rate and 11.4% of people at risk of poverty. Savinjska region had a regional GDP of 16.455 €/resident, 13.9% registered unemployment rate and 15% of people at risk of poverty.^32^ Lower socio-economic factors are usually associated with longer intervals.^12,33,34^ In 2014 Savinjska and Osrednjeslovenska region had the same age-standardized prostate cancer incidence rate (99.2 vs. 100.1). Savinjska region had a higher standardized death rate (574.8 vs. 463.0) and age-standardized death rate due to neoplasms (231.4 vs. 189.4) than Osrednjeslovenska region.^35^ Taking all these data into account, it is difficult to explain the shorter primary care interval in the Savinjska region.

In this study we also aimed to explore potential associations between the duration of primary care interval and the 5-year survival rates among prostate cancer patients in Slovenia. As expected we found worse 5-year survival in older patients, with higher cancer stages, lower education and more comorbidities.^25^ Survival was not influenced by the region where the GP worked. We found a statistically significant difference in 5-year survival in patients with lower access to laboratory tests and abdominal ultrasound by their GP, and in symptomatic patients compared to those with a first contact due to a detected elevated PSA level. Other research has also confirmed that the quality and speed of the diagnostic process is influenced by a number of factors beyond the diagnostic skills of individual clinicians, such as the tests and services available to them, time constraints to consultation duration and the quality of doctor-patient communication.^10^

The use of the Slovenian Cancer Registry database enabled a comprehensive analysis as a substantial number of patients diagnosed with prostate cancer in 2014 were included. This large cohort provides robust statistical power and improves the generalizability of the study results. By using registry information, all patients diagnosed with prostate cancer in 2014 were identified and included in the study, minimizing potential selection bias and ensuring a representative sample. The limitation was the 58% response rate. As part of the study, GPs were encouraged to review both paper and electronic patient records, including discharge letters, to ensure comprehensive and accurate reporting. This approach aimed to reduce recall bias and increase the validity of the results.

The retrospective nature of the study, relying on established diagnoses and historical data, brings inherent limitations. In particular, the study focused on the doctor and primary care interval rather than the patient interval, which could affect the accuracy of reported symptoms and timing. Despite efforts to minimize recall errors, incomplete information in some questionnaires may have led to information bias, particularly with regard to the duration of doctor and primary care intervals. This bias could underestimate the actual intervals as it is based on fragment-ed or incomplete patient records. The change of GP chosen by patients between diagnosis and data collection, coupled with the variability of medical record systems, posed a challenge in accessing complete and consistent data. Paper records were frequently relied upon, which were often incomplete and difficult to decipher, while electronic records experienced compatibility issues, further complicating data retrieval and potentially leading to missing or inaccurate information. The presence of missing data leads to uncertainty about the actual performance or recording of diagnostic procedures, such as digital rectal examinations, which could affect the completeness and reliability of results. The same applies to shared decision-making. Clinical practice guidelines on prostate cancer screening using the PSA test have clearly recommended that clinicians practice shared decision making - a process involving clinician-patient discussion of the pros, cons, and uncertainties of screening. However, studies have shown that most men have never engaged in shared decision-making conversations with a healthcare provider about PSA testing.^36,37^ In our study, shared decision-making was not recorded in the GP's medical records, but we cannot say with certainty that it was not carried out either.

This study provides a comprehensive examination of early management of prostate cancer patients within the primary healthcare system in Slovenia and offers valuable insights into diagnostic timelines and their impact on patient outcomes. The significant correlations found between the duration of intervals, 5-year survival rates and accessibility of laboratory diagnostics emphasize the crucial role of timely and comprehensive diagnostic testing in improving prognosis. From a clinical perspective, these findings underscore the importance of ensuring adequate resources and infrastructure for diagnostic testing in primary health care centres to minimize delays in diagnosis and optimize patient care. In addition, the observed regional differences in diagnostic intervals highlight the need for targeted interventions to address inequalities in access to healthcare and improve diagnostic efficiency in different regions. From a research perspective, these findings provide a basis for further investigation of the underlying factors influencing diagnostic processes and the effectiveness of interventions aimed at reducing diagnostic delays. Future research efforts should prioritize the validation of these findings through longitudinal studies and comparative analyses, while exploring new strategies to increase diagnostic efficiency and improve patient outcomes in prostate cancer. This study also emphasizes the need for further investigation of the factors that influence an individual's decision to seek primary health care services, particularly those beyond the scope of the variables included in the current model.
